# Making Robust Policy Decisions Using Global Biodiversity Indicators

**DOI:** 10.1371/journal.pone.0041128

**Published:** 2012-07-18

**Authors:** Emily Nicholson, Ben Collen, Alberto Barausse, Julia L. Blanchard, Brendan T. Costelloe, Kathryn M. E. Sullivan, Fiona M. Underwood, Robert W. Burn, Steffen Fritz, Julia P. G. Jones, Louise McRae, Hugh P. Possingham, E. J. Milner-Gulland

**Affiliations:** 1 Department of Life Sciences, Imperial College London, Berkshire, United Kingdom; 2 School of Botany, the University of Melbourne, Victoria, Australia; 3 Institute of Zoology, Zoological Society of London, London, United Kingdom; 4 Environmental Systems Analysis Lab, University of Padova, Padova, Italy; 5 Department of Animal & Plant Sciences, University of Sheffield, Sheffield, United Kingdom; 6 Department of Mathematics and Statistics, University of Reading, United Kingdom; 7 International Institute for Applied Systems Analysis (IIASA), Laxenburg, Austria; 8 School of Environment, Natural Resources and Geography, Bangor University, Bangor, United Kingdom; 9 The Australian Research Council Centre of Excellence for Environmental Decisions, The University of Queensland, St Lucia, Queensland, Australia; University of Durham, United Kingdom

## Abstract

In order to influence global policy effectively, conservation scientists need to be able to provide robust predictions of the impact of alternative policies on biodiversity and measure progress towards goals using reliable indicators. We present a framework for using biodiversity indicators predictively to inform policy choices at a global level. The approach is illustrated with two case studies in which we project forwards the impacts of feasible policies on trends in biodiversity and in relevant indicators. The policies are based on targets agreed at the Convention on Biological Diversity (CBD) meeting in Nagoya in October 2010. The first case study compares protected area policies for African mammals, assessed using the Red List Index; the second example uses the Living Planet Index to assess the impact of a complete halt, versus a reduction, in bottom trawling. In the protected areas example, we find that the indicator can aid in decision-making because it is able to differentiate between the impacts of the different policies. In the bottom trawling example, the indicator exhibits some counter-intuitive behaviour, due to over-representation of some taxonomic and functional groups in the indicator, and contrasting impacts of the policies on different groups caused by trophic interactions. Our results support the need for further research on how to use predictive models and indicators to credibly track trends and inform policy. To be useful and relevant, scientists must make testable predictions about the impact of global policy on biodiversity to ensure that targets such as those set at Nagoya catalyse effective and measurable change.

## Introduction

In response to the general failure to meet the Convention on Biological Diversity (CBD) goal to reduce the rate of loss of biodiversity by 2010, the October 2010 Conference of the Parties to the CBD agreed to a Strategic Plan with new targets for biodiversity conservation until 2020 [1], [2]. This Plan aims to inspire action to halt the ongoing loss of biodiversity through the development of national biodiversity strategies, targets and action plans [1]. A set of CBD indicators for assessing and communicating trends in seven focal areas, including biodiversity [3], [4], were used to assess the 2010 goals, and a similar set of indicators are suggested for assessing progress towards the new targets [1], [5], [6]. A key reason for the development of global biodiversity indicators was their potential to evaluate actions and develop understanding of underlying processes and drivers of loss [4], [7], [8], but this is virtually never done [7], [9]. Instead, indicators have principally been used to track the status of and trends in biodiversity and drivers of loss, from which the impact of actions is inferred [3], [10]. A Responses-Pressures-State-Benefits framework is starting to be used by the CBD for presenting linked sets of biodiversity indicators [11], [12]; this implies causative links between changes in groups of related indicators, but without an explicit model of the mechanisms underlying these interactions.

**Figure 1:**
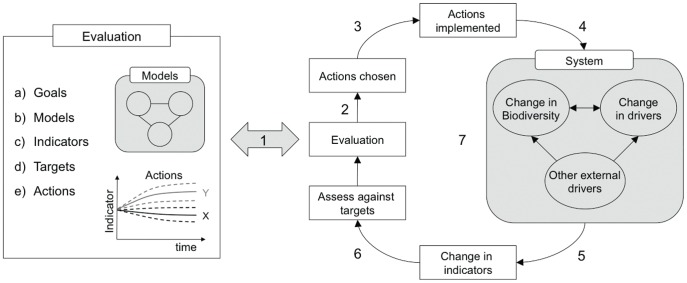
The indicator-policy cycle, a framework for using indicators to inform policy: Evaluation of the problem at hand involves defining broad goals of policy, developing models of the system, selecting indicators that reflect the changes of interest, defining specific targets that can be measured with the indicators, and defining a set of actions or policies to achieve the targets, which are assessed using the models. After evaluation, actions are chosen and implemented, with resultant change on biodiversity and drivers of loss, which may also be affected by other external drivers unrelated to the actions; change is monitored with the indicators and assessed against the targets, leading to re-evaluation. Key sources of uncertainty and potential failure throughout the cycle are numbered and discussed in detail in Table S1: 1) assumptions in the evaluation process; 2) the link between evaluation and selection of actions; 3) the link between selection and implementation of actions; 4) the impact of the action differing from the anticipated impact; 5) the link between biodiversity change and indicator change; 6) the link between indicator change and target assessment; and 7) mismatches in temporal and spatial scales throughout the cycle.

To make sensible and robust policy decisions, an explicit understanding of the linkages between action and outcomes is needed. This requires the impacts of policies to be projected forward using models, ideally as part of an adaptive decision-making process that includes defining targets and monitoring the results, which we term an “indicator-policy cycle” (Figure 1). Models can be used by policy-makers to assess the impact of their decisions, and to learn and evaluate both our understanding of the relationship between policy action and environmental change, and the appropriateness of the indicators for measuring change. Indicators are a key link in the cycle, as the means by which policy outcomes are communicated and evaluated. However, there has been very little evaluation of the robustness of indicators in representing underlying biodiversity trends of interest, with Branch et al. [13] and Fulton et al. [14] providing notable exceptions.

In this paper we first outline the indicator-policy cycle, and highlight the areas of strength and weakness in current applications of the cycle. We then illustrate how the CBD’s global biodiversity indicators can be used with modelling to predict the impacts of alternative policies, using two case studies: a comparison of protected area policies for African mammals, assessed using the Red List Index (RLI), an index of extinction risk for species of plants and animals [15]; and an assessment of the impact of a reduction in bottom trawl fishing, measured using the Living Planet Index (LPI), a composite of time series of vertebrate abundance and biomass [16]. The aim is not to provide a detailed and comprehensive assessment of the impacts of the alternative policies. Rather, the aims of these case studies are first to demonstrate how biodiversity indicators can be used predictively to evaluate the impacts of policy, and secondly to test the ability of the indicators to represent biodiversity trends and to assess the effects of underlying data biases on the trends they predict. The case studies provide a test of the ability of two of the most important and best-developed CBD indicators to demonstrate progress towards the CBD’s 2020 targets.

We chose to model protected areas (PAs) in the first case study, as they form the cornerstone of conservation, recognised in Target 11 of the headline 2020 CBD targets, which calls for at least 17% of terrestrial areas to be placed in effective and well managed protected areas by 2020 [1], [5]. However, the extent of PA coverage alone gives little information on how well PAs are performing in protecting biodiversity [17]. Many PAs perform poorly in maintaining biodiversity within their boundaries; for example, Craigie et al. [18] found on average a 59% decline in population abundance of large mammals between 1970 and 2005 in 78 African PAs, with considerable variation between regions and countries. We chose to test the effects of policy on the RLI as PAs are commonly instituted in order to protect threatened species. We modelled the impact of four policies on trends in vertebrate numbers in sub-Saharan Africa and on the RLI, and then evaluated to what extent the RLI reflected these modelled trends. We compared 4 policy scenarios: 1) business-as-usual; 2) expanding PAs to 17% of the terrestrial area of each country; 3) improving management effectiveness in current PAs; and 4) expanding PAs to 17% and improving management effectiveness.

In the second case study, we modelled the impact on biodiversity of two alternative policies, halving or halting bottom trawling, in six ocean systems using 10 ecosystem models, and calculated the ensuing changes in the LPI. Reduction in bottom trawling is one potential policy action that could be implemented in response to the CBD’s 2020 Target 6: “By 2020 all fish and invertebrate stocks and aquatic plants are managed and harvested sustainably, legally and applying ecosystem based approaches…*”* [1]. We chose to use the LPI in this case because it is more appropriate for representing trends in common or abundant species such as may be most affected by large-scale trawling. We compared the trends in the LPI with modelled trends in abundance of species assemblages.

### The Indicator-policy Cycle

We argue that indicators should be used within an indicator-policy cycle (Figure 1) that places monitoring with biodiversity indicators within an decision analytic framework [19], [20], drawing on adaptive management [21], [22], management strategy evaluation [23], and optimal monitoring [24], [25], where monitoring is explicitly linked with action and learning. These approaches are currently typically applied only at local to regional scales and to single species management [24], [26], [27], although the cycle in Figure 1 has parallels with emerging approaches to using ecosystem-based indicators to manage and monitor the effects of fishing [14], [28], [29], [30]. The first stage comprises *evaluation* of the problem at hand:

- *Define broad policy goals*: For example five strategic goals for 2020 are identified in the CBD Strategic Plan, relating to pressures on biodiversity, its status, benefits gained, mainstreaming biodiversity and implementation [1], [31].
- *Develop a model or models* of the system to understand system dynamics and predict the impacts of alternative actions, based on an understanding of underlying processes. Ideally models will be quantitative, with an estimate of uncertainty in the predictions; however qualitative conceptual and expert-based models of how actions may affect indicators can provide a basis for informing policy choices [32], [33], [34], [35]. Our case studies differ in the types of models used: the first uses statistically derived trend data for African mammals within protected areas; the second uses process-based models of marine systems.
- *Select indicators* that measure trends of interest. Indicators are needed as proxies for communicating the complex realities of biodiversity change. Much has been written on indicator selection and assessment [8], [14], [20], [36], but the crucial need is for indicators to be tested, in order to assess their ability to detect relevant trends and measure progress at appropriate scales, and to reflect change in response to policies relative to the impacts of other drivers [14], [29]; this is the focus of the case studies below.
- *Narrow the broad policy goals to specific targets* or milestones that are measurable using the indicators, such as a specific threshold value of an indicator [37], (e.g. the CBD 2020 Target 11: at least 17% of terrestrial areas within protected areas (PAs), measured by PA coverage [5]), or a predefined meaningful rate of change in the indicator [38] (e.g. CBD Target 12: by 2020, the extinction of known threatened species has been prevented and their conservation status has been improved and sustained [5], which implies an increasing Red List Index (RLI)). Whether or not a target has been met can be used as a trigger for action.
- *Define a set of actions* that can be implemented to achieve the targets and use the model(s) to *make predictions* about the potential impact of each potential action on biodiversity. The predictions should quantify the effect on both the underlying system of interest and the biodiversity indicators that will be used to monitor system changes; we demonstrate this below using two case studies. This information can then be used to improve monitoring, in turn leading to learning that can improve models. The impact of uncertainty on predictions should be quantified and presented explicitly [7].

Actions are selected and then implemented, with direct and indirect impacts on biodiversity and drivers of loss. Direct effects might include the reduction of threats inside a newly implemented protected area; indirect effects include displacement of fishing activity from a marine protected area [39]. Other extrinsic drivers can cause change unrelated to the actions, such as environmental factors (e.g., drought, climate change), or political or macro-economic change, making the effects of policy change difficult to isolate [74]. If indicators are appropriate, they should change proportionately to the changes in biodiversity and drivers. The indicators are then assessed against the targets. Finally, the models, indicators, targets and management actions are subjected to iterative evaluation and reviewed in light of new monitoring data.

There are many points at which failure or uncertainties can occur within the indicator-policy cycle. We identify seven potential points of failure, numbered in Figure 1; examples of each, how they can be addressed and by whom, are listed in the supplementary material (Table S1). A major source of uncertainty in the indicator-policy cycle is the relationship between the indicators and the underlying trends of interest, which has two components: the design of the indicator, and the quality or bias in the data used to estimate it; we explore these in the case studies below. Trade-offs inevitably exist: an indicator might be a good proxy for biodiversity but the data needed are very difficult or expensive to collect; alternatively an indicator might be readily estimated with available data, but is a poor proxy for biodiversity [13], [17], [40].

## Results

### Case study 1: The Effects on Mammals of Policies for Protected Area Management in Africa

**Figure 2:**
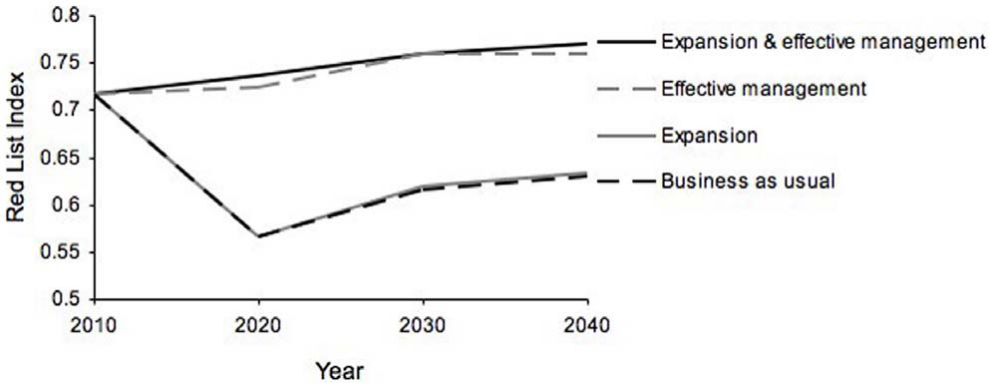
Changes over time in the Red List Index (RLI) under four policy scenarios for protected area management in Africa: 1) business-as-usual, 2) expansion of terrestrial PA network to 17% of each country, 3) improved management effectiveness of PAs, and 4) expansion to 17% with improved management effectiveness. The RLI was calculated for 53 species of large mammals in sub-Saharan Africa; an increase in the RLI means a reduction in the average extinction risk of this set of species.

The predicted effect of changes in PA policy, as reflected by changes in the RLI, suggested that without effective management, expanding protected areas provided negligible benefit over business-as-usual (BAU). Improving management effectiveness, and thus stopping declines in abundance, provided much greater benefits to the study species than just expanding PAs where declines continue (Figure 2). Changes in the RLI were primarily driven by population declines of threatened species in ineffective PAs in Central and West Africa. Most of the study species were not highly threatened and policy changes tended not to affect their Red List categories.

Under effective management of current PAs, the RLI gradually increased, showing a general improvement in species status over time (Figure 2). Under both BAU and PA expansion, the RLI declined rapidly at first, but later started to increase. This was due not to improved species status but to shifts in the relative contribution of populations that were increasing or decreasing to the overall trend in population size, and the relatively short timeframe over which declines are assessed under the Red List criteria. Most of the threatened species had some declining and some stable or increasing populations. As the declining populations became small, they contributed less to the overall trend for the species as a whole, leading to species being downlisted under Criterion A of the IUCN Red List, which relates to population declines [41]. Secondly, the IUCN Red List criteria assess declines over a period of 3 generations or 10 years, whichever is larger up to 100 years. Because there were few very long-lived species in our dataset, most species had an assessment of period of between 10 to 20 years. Consequently, large early declines were ‘forgotten’ by later assessments, and the RLI increased. Nevertheless, although the RLI was not particularly sensitive to the policies that we examined, and trends in the indicator did not exactly relate to changes in species abundance, the indicator was able correctly to reflect the relative impacts of policy changes on biodiversity (as represented by mammal population trends).

Another CBD indicator is PA coverage itself. Using PA coverage as an indicator would lead to PA expansion being chosen as the preferred option over improved management effectiveness, because the indicator does not account for the performance of PAs. A new CBD indicator of PA effectiveness is currently under development [4]. Under our PA expansion scenario, average PA coverage was 19% (ranging from 17% to 36%); currently, 29 of the 41 study countries have less than 17% of their land area in PAs (mean PA coverage 12.5%, from 0 to 36% [42]). Even though the RLI is a relatively coarse indicator, it is more effective at representing the effects of policies on biodiversity than PA coverage [41].

### Case study 2: The Effect of Bottom Trawling on Marine Biodiversity

The LPI showed muted and often negative responses to the policies of halving and halting bottom trawling (Table 1), although the total vertebrate biomass in the models increased in each ocean system for both scenarios, with the exception of the North Pacific when trawling effort was halved (Table 1). The model results were driven by the relative increase in biomass of species that had been targeted by bottom trawling or caught as by-catch, and by the resultant trophic interactions. The composition of the data used to construct the LPI affected its overall trend. The LPI datasets within the study regions are dominated by seabirds and fish (Table 2). Biomass trends of seabirds varied by region (Table 1): they decreased in the North Sea [43] and Mediterranean Sea [44] models, where they are highly reliant on discards for food, and in one of the South Atlantic models [45], due to increased competition after a release of fishing pressure on predatory fish. In the Caribbean, seabirds increased with one of their main prey species, which was released from direct fishing pressure. This range of responses in seabirds alone made the interpretation of trends in the LPI difficult.

**Table 1:** The impact on the Living Planet Index (LPI) of halving or halting bottom trawling in six ocean systems, based on biomass trends in modelled vertebrate groups represented in the LPI.

Ocean system	LPI: Halve	LPI: Halt	Vertebrate biomass: halve	Vertebrate biomass: halt	Mammals	Seabirds	Sea turtles	Sharks & rays	Other fish
**Global**	−2.5%	−1.3%	0.7%	2.0%	∼	∼	∼	∼	∼
**North Pacific**	−5.6%	−0.7%	−0.1%	1.4%	∼	∼	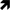	∼	∼
**North & Baltic Seas**	2.7%	−3.3%	2.2%	5.1%	∼	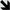		∼	∼
**Mediterranean & Black Sea**	−0.7%	−0.8%	1.4%	3.4%	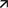	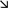		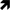	∼
**South Pacific**	3.6%	5.3%	3.8%	7.5%	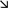	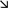	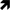	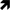	∼
**South Atlantic**	−4.7%	−3.8%	0.1%	0.3%	∼	∼		–	∼
**Caribbean & Gulf of Mexico**	−1.5%	−1.3%	0.8%	1.5%	–	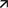	–	∼	∼

Columns show the % change in the LPI 30 years after implementation of the policies, and the % change in total vertebrate biomass; and for each taxonomic group, the general biomass trends for different species groups from the Ecopath models from halting bottom trawling (the general biomass trends per group were typically stronger under a halt in bottom trawling than under a halving of effort); symbols: – <5% change, 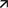 >5% increase, 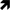 >20% increase, 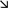 >5% decrease, 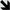 >20% decrease, ∼ mixed: different responses were seen across models, species and/or functional groups, blank denotes that the group was not modelled.

Fish were the other group that dominated the LPI (Table 2). The response of fish populations to the cessation of bottom trawling was mixed (Table 1). Whether more predator or prey fish species were included in the LPI, and how each was affected directly or indirectly by the policy, affected indicator behaviour. For example, in the Mediterranean [44], an end to trawling was predicted to have a very positive impact on the biomass of hake (directly targeted by fishing), flatfishes and rays (increases of 69%, 52% and 112% respectively). The LPI for the region included trends for two flatfish species and the hake, but did not include trends for ray species or the key prey groups that decreased as a result of predation by increased hake and elasmobranch populations. By contrast, the LPI in the Mediterranean includes 16 bird species; seabirds as a group were expected to decline by just under 20% based on a reduction in discards alone (other threatening processes were not included in the model).

**Table 2:** The modelled species groups used to calculate the LPI for each region.

Ocean system	No. spp	Mammals	Seabirds	Sea turtles	Sharks & rays	Other fish
**Global**	377	10%	36%	3%	3%	49%
**North Pacific**	142	19%	27%	3%	3%	48%
**North & Baltic Seas**	85	0%	46%	0%	4%	51%
**Mediterranean & Black Sea**	28	7%	57%	0%	11%	25%
**South Pacific**	44	2%	34%	7%	0%	57%
**South Atlantic**	86	6%	42%	0%	1%	51%
**Caribbean & Gulf of Mexico**	26	4%	35%	19%	8%	35%

The number of species used in the LPI for each system, and the percentage of species in each group that contributed to the LPI database.

## Discussion

There are many potential barriers to successfully halting biodiversity loss; the complexity and scale of the problem is enormous. The difficulties in collecting sufficient data to understand the system and monitor trends emphasises the need to have a clear understanding of the changes of interest and to evaluate the capacity of a suite of indicators to detect them based on improved data and scientific understanding. Recent initiatives such as GEO-BON (The Group on Earth Observations Biodiversity Observation Network) for monitoring global biodiversity [46], and IPBES (the Intergovernmental Science-Policy Platform on Biodiversity and Ecosystem Services [47]) will contribute to bridging the gap between multiple sources of information, research and policy [48]. However improved data and understanding are not enough unless actions are also set within a framework that enables informed decisions to be made, and their impact on biodiversity evaluated both *a priori* and through ongoing monitoring. Such a framework needs to include evaluation of the validity of the indicators used by policy-makers to assess progress towards their targets.

The two key questions we posed with our case studies were: do the policies have the expected impact on biodiversity? And do the chosen indicators reflect this impact? In the case of the African PAs, both answers were yes: species trends improved with effective management, though the magnitude of the difference between scenarios that included or excluded management effectiveness, and the small impact of PA expansion without increased management, were perhaps surprising. The RLI clearly reflected the qualitative differences between policies, despite its counterintuitive trajectory under PA expansion and BAU. However the modelled impact of a cessation of bottom trawling on biodiversity was mixed: some species and groups increased in biomass, others decreased (e.g. seabirds decrease in abundance in several of the modelled systems). In an ideal system, all species would be monitored; in reality this is not the case. Because more data were available on seabirds than other taxonomic groups, there was a corresponding, disproportionately large impact on the LPI from seabird trends due to data bias. As a result, the overall trends shown in the LPI were not representative of all the populations in the ecosystem, which showed largely positive trends. In any system where policy impacts vary across species, particularly when there are negative impacts on some groups, the trends in indicators will be sensitive to the composition of data that feed into them, supporting the need for testing indicators.

The response to policy of both the species of interest and the indicators reflected the modelling approach used and the species included in the indicators. In the African mammals example, we assumed that all species would benefit from improved management effectiveness, which simplified the trends that the indicator had to reflect; this assumption can be justified by similar threatening processes faced by many African mammals, such as over-hunting and habitat loss [18], [49]. It is also likely that species interactions would affect trends in some species; for example negative impacts on some ungulates might be expected due to increased abundance of predators [50]; such interactions were not included in our simple trend-based models. By contrast, in the Ecopath models, trophic interactions were included explicitly, reflecting both direct and indirect effects of policies, such as increasing predator numbers and resultant decreases in prey and in competitors. However, the Ecopath models did not include impacts of threats other than fishing or recent trends in abundance, and therefore may underestimate the risk of extinction for many species. For example, while seabird abundance declined in several models, this was due to reduction in food availability from by-catch, and not other threats such as loss of nesting sites or predation. The Ecopath models were not designed with the intention of modelling threats faced by individual seabird species, but rather modelled seabirds as a functional group within a trophic web. Species-specific threat modelling in both case studies would better account for such effects, but would be difficult when dealing with large numbers of species. However, the aim of this study was not to provide a comprehensive scenario analysis of the impact of halting trawling or expanding PAs in Africa, but to illustrate how CBD targets and indicators can be evaluated within a predictive modelling framework.

Our results show the importance of having an indicator with a robust relationship to the underlying system dynamics. Both indices relate to extinction risk: the LPI through one symptom of risk, geometric mean abundance, and the RLI by using a suite of symptoms of risk [51]. The RLI provides a relatively coarse-scale assessment of extinction risk based on movement between the IUCN Red list categories that classify relative extinction risk of species based on threshold values for decline and abundance [52]. Thus movement from one threat category to another requires either a substantial change in abundance or trends, or for the species to already be very close to a threshold value. Consequently, the RLI would be unlikely to differentiate between the marine trawling policies, which have large effects for non-threatened species and mixed (some negative, some positive) for more threatened species.

The trawling case study demonstrates the value of indicator assessment to diagnose the basis for poor indicator performance. While the potential effects of taxonomic bias in indicators has been discussed elsewhere [4], [16], [53], process-based models such as this explicitly demonstrate the quantitative impacts of the underlying data and the specific assumptions of predictive models on indicator performance [13], [14]. Previous modelling has suggested that biomass indicators are amongst the most robust in detecting the effects of fishing, particularly for functional groups and higher trophic levels [14], [29], although aggregating data can mask trends because increases in some groups balance decreases in others [29], [54], or result in counter-intuitive responses to policy. These previous results, combined with the case study presented here, suggest that single indicators do not easily represent policies with complex ecosystem effects [13]. Indeed, the approach of ecosystem-based fisheries science is to use a suite of indicators together with a set of validated alternative models to explore plausible scenarios of change [14], [28], [30].

Currently global biodiversity indicators have been used only to report on trends, effectively comprising only two stages of the indicator-policy cycle illustrated in Figure 1. Adaptive management in general has been applied in limited ways at the local level [22]. Indicators have rarely been used to report against explicit targets or baselines, nor have they been linked into evaluation of the effectiveness of action. Most model-based predictions of policy effects have not used biodiversity indicators that can be readily monitored, instead relying on metrics such as mean species abundance and projected number of species extinctions [7], [9], [34]. Evidence-based modelling, both predictive (using scenarios and counterfactuals) and retrospective (using statistical models), allows different processes and causal relationships to be understood [32], [55], [56] and is essential to improving the value of biodiversity indicators for decision-making. If conservation scientists are to influence policy, they must be bold enough to make predictions and give advice that aids decision-makers, or they will remain peripheral to important decision-making processes.

## Materials and Methods

### Case study 1: The Effects on Mammals of Policies for Protected Area Management in Africa

We modelled the impact on a key CBD indicator, the Red List Index (RLI) [15], of continental-scale policies for African terrestrial PAs based on current trends in vertebrate abundance: 1) business-as-usual; 2) expanding PAs to 17% of the terrestrial area of each country; 3) improving management effectiveness in current PAs; and 4) expanding PAs to 17% and improving management effectiveness (for more details, see [41]). The aim of this study was not to provide a detailed analysis of the effects of these policies on biodiversity, but instead to assess whether the RLI effectively captured realistic trends in biodiversity caused by a policy change. The analyses comprised a series of steps, first to establish estimates of population size, distribution and current rates of decline for each species, then the application of the policy scenarios, and finally, resultant changes in species abundance under the scenarios were fed into the Red List Index. For more details on methods, further scenarios and sensitivity analyses, see [41].

Our case study comprised 53 mammal species in 41 countries in four regions, East, Southern, West, and Central Africa (Table S2). Trends in mammal abundance in PAs vary greatly by region; for example, declines are greatest in West and Central Africa, with more moderate declines in East Africa, and increasing populations in Southern Africa [18]. Country-level estimates of population size for each species inside and outside of protected areas were collated or estimated from IUCN species survival commission and other publications (see Table S2 for sources) [41].

For each species in each region, inter-annual trends in population sizes within protected areas were estimated from time series as described in [18]. The time series varied in length and the amount of data; where six or more data points were available, a GAM was used to estimate inter-annual trends following [16]; where there were fewer data, inter-annual rates of change were estimated between consecutive years or linearly interpolated for non-consecutive years, and averaged across years [18]. Where multiple estimates of trends were available within a region, such as several PAs in one country or across several countries, the geometric mean of population trends was used to produce a regional trend per species in PAs. Currently no comprehensive trend data exist for the study species outside PAs, although empirical data suggest lower densities of mammal species outside PAs than inside in Tanzania [57], [58], supported by expert opinion on African tropical forests [49]. In the absence of trend data, we assumed that population trends for all species would be 25% worse outside PAs than inside PAs. Therefore, positive trends were decelerated by 25% and negative trends accelerated by 25%. Sensitivity analyses showed the qualitative results to be robust to the assumed difference in trend between populations inside and outside PAs [41].

The impact of each policy scenario on trends in abundance of the study species was modelled over a 30-year period, assuming constant annual trends and immediate implementation. We modelled four scenarios:

#### Scenario 1: Business as usual (BAU)

Current population trends in PAs for each species in each region were applied to populations inside existing PAs (from the World Database on Protected Areas in 2010 [42]). Trends outside PAs were assumed to be 25% worse than those inside PAs.

#### Scenario 2: Expand terrestrial PA coverage to 17% of each country

We used the conservation planning software Marxan [59] to select 25×25 km gridcells to add to the current PA network, based on habitat suitability models for the target species [60]. We used country-level targets of 17% of each country. The continental-level target for species was between 5% and 100% of the total area of suitable habitat [60]. We assumed that the suitable habitat in the added PAs was occupied (thus potentially over-estimating the positive impacts of PA expansion due to commission errors) and had the same population density as the current PAs. The populations in the newly-added PAs were therefore a function of the area of suitable habitat and the density of the species within the current PAs, and was subtracted from the formerly unprotected populations to ensure that the total population of the species within a country did not increase immediately upon adding PAs. The population trends for each species in each region applied to populations inside and outside PAs were the same as those used in BAU (Scenario 1).

#### Scenario 3: Improved management effectiveness in Pas

No consistent data exist on the impact of effective management within PAs across Africa. PAs in Southern Africa are considered to be most effectively managed [18]. Therefore we assumed that populations in effectively managed PAs would experience the same annual rate of increase as the average across all species in Southern African PAs (+1.8% [18]), except for those that already had a more positive annual trend, which was assumed to stay constant. A sensitivity analysis showed that the value of the assumed trend in effectively managed PAs had no effect on the relative impacts of the policies [41]. Population trends outside PAs were the same as those used for in BAU (Scenario 1).

#### Scenario 4: Expand PA coverage to 17% of each country and increase management effectiveness of Pas

The areas added to the current PA network were the same as those described above in the expansion scenario (Scenario 2), with the same corresponding redistribution of populations between PAs and non-PAs. The population trends inside PAs were the same as those used in the effectiveness scenario (Scenario 3), based on current trends in Southern Africa. Trends outside PAs were the same as BAU (Scenario 1).

**Table 3:** The ten Ecopath models used to simulate the policies of ending and halving bottom trawling, the stated objectives in the studies in which the models are described, the number of functional or taxonomic groups each model contained, the number of these groups represented in the LPI, the fraction of fishing fleets that were bottom trawl-based and thus affected by the policies, and the ocean system the region lies in.

Model Regionand reference	Model objective	Groups in Ecopathmodel	Groupsin LPI	Bottom trawlfleets/totalfishing fleets	OceanSystem
Central Gulf ofCalifornia [70]	To characterize the trophic relationships and biomass flowpaths; to learn the role of some functional groups,particularly of discards, in the ecosystem	27	7	1/4	North Pacific (temperate)
East ChinaSea [71]	To examine possible mechanisms leading to jellyfish bloomsand the impact of these blooms on fishery resources	45	11	1/6	North Pacific (temperate)
Western and Central Aleutians [69]	To examine the decline in the western stock of Steller sealions, Eumetopias jubatus	40	21	1/6	North Pacific (temperate)
North Sea [43]	To quantitatively describe the ecological and spatial structureof species assemblages of the North Sea ecosystem; and tocalibrate the dynamic responses of the modelled systemby comparison with observed historical changes	68	27	4/12	North & Baltic Seas (temperate)
Northern AdriaticSea [44]	To analyse the trophic structure of the system, identify the keytrophic groups, and assess anthropogenic impacts onthe ecosystem	34	9	2/6	Mediterranean & Black Sea
Great BarrierReef [72]	To identify the effects of the major fisheries in each of thecomponent systems, and the possible confounding effects ofindependently developed fisheries management plans	32	8	1/3	South Pacific (tropical)
NorthernBenguela [73] *	To construct an improved, updated, dynamic ecosystem modelof the trophic flows of the northern Benguela, to facilitate thedevelopment and evaluation of multispecies managementtechniques for the marine resources of Namibia and possiblythe entire Benguela	26	2	1/8	South Atlantic (tropical)
SouthernBenguela [45] *	To identify data gaps and imbalances that result frominconsistencies between various stock assessments; …to assess how observed differences or similarities in abundance, catches and dietary composition could affect overall trophic functioning, focusing on the pelagic part of the southernBenguela ecosystem	27	11	1/6	South Atlantic (tropical)
West FloridaShelf [68], [74]	“to evaluate the potential effects of shading by phytoplanktonblooms on community organization”“The general questions addressed in this study were: (1) Arethere multiyear trends in water transparency over the WestFlorida Shelf? (2) What proportion of the overall primaryproduction on the West Florida Shelf is made up by microphytobenthos?(3) What broad community effects might result from nutrientenrichment and phytoplankton blooms?”	59	6	1/11	Caribbean & Gulf of Mexico (tropical)
Gulf of Mexico,AlvaradoShelf [75]	“to integrate in a coherent way knowledge about the system, tolearn more about the structure and function of the system, andto help to understand the ecosystem function”	40	6	1/1	Caribbean & Gulf of Mexico (tropical)

* The models used for Northern and Southern Benguela are updated versions of the published ones, provided by Lynne Shannon (Southern Benguela) and Jean-Paul Roux (Northern Benguela), while the model for the Gulf of Mexico was provided by V.H. Cruz-Escalona.

#### Calculating the RLI

From the population projections in each scenario, each species was assigned to a Red List category at decadal intervals using the IUCN Red List Criteria version 3.1 [61] criteria A and C (reflecting decline rate and population size respectively), using generation times from the PanTHERIA database [62]. The trends for criterion A were based on the current inter-annual trends and species-specific generation time, and thus estimated trends for a time period that include both past and future trends (Criterion A4) or future time period alone (Criterion A3). The population size (IUCN Criterion C) at each modelled assessment was estimated using the total projected abundance as a result of the policies in each scenario; for simplicity’s sake the sub-criteria for Criterion C were not applied, and all individuals were assumed to be mature. The Red List index was calculated for each scenario at decadal intervals [15].

### Case study 2: The Effect of Bottom Trawling on Marine Biodiversity

Bottom trawling has significant effects on marine biodiversity [63], and there have been calls for the practice to be halted [64]. We simulated the effects of halting and halving bottom trawling on marine ecosystems using Ecopath with Ecosim (EwE), a modelling suite for constructing food-web models of marine systems [65]. In EwE, a food-web is represented as a set of mass/energy flows connecting the model groups, which can represent populations, functional and taxonomic groups, or life-history stages. Each model group is characterized by the total biomass of the organisms composing it, their diet preference, production and consumption rates, and so forth. Two main steps are required to simulate the biomasses of such groups over time. First, a static, mass-balanced food-web model is constructed. A model is mass-balanced if the flows entering any given group (consumption) equal the sum of its unassimilated consumption, production and respiration, and if production is equal to the sum of all the possible mortality sources (predation, fishing, other sources), of net migration fluxes and of biomass accumulation in that group. The mass-balance condition is useful to constrain the uncertainty in model parameters, ensuring that they are mutually consistent and that mass/energy is conserved. As a second step, the static model is used as an initial condition to simulate changes in the biomass of the model groups over time, through a system of differential equations. Biomass variations can result from “top down” processes, such as biomass removal through fishery catches and predator-prey interactions, and “bottom up” processes; for example, the modeller can force primary productivity or simulate how it is affected by nutrient loads. Other bottom up processes such as the effects of temperature and climatic variability are more difficult to simulate, because EwE is focused on biomass flows and trophic interactions, especially in higher trophic levels. Because complex trophic interactions are taken into account, the cascading effects of fishing across the food-web can be simulated. Therefore, EwE modelling allows the direct as well as indirect effects of fisheries to be quantified [65]. Direct effects of bottom trawling may include fishing pressure on the target species as well as by-catch and discarding practices (e.g. of benthic organisms caught by bottom trawling), while indirect effects include trophic interactions [63].

We used ten Ecopath models from six ocean systems (Table 3), with the goal of covering different ecosystems worldwide. The models were selected based on model quality, documentation and data availability; key information such as data sources, input data and parameter estimates, and predator-prey and diet matrices for each model can be found in the relevant references (Table 3). Only models that included species in the LPI dataset and with separate bottom trawling fleets were used. The aims of each model varied to some extent (Table 3), but generally the models were built with the purpose of better understanding the trophic interactions of the systems, and the impacts of anthropogenic pressures such as fishing.

Each model was run for 20 years prior to policy implementation to allow the biomass trajectories to stabilise, and for a further 50 years after the policy implementation to allow for recovery of longer-lived species and for resulting biomasses to stabilise. The Ecopath models did not cover all areas of each ocean system modelled; rather we treated the models as samples of each system, and extrapolated trends in groups from each model across the ocean systems, similar to [66]. This assumed that fishing pressure and the subsequent reduction in bottom trawling were uniform across a given ocean system.

The impacts of the policies on functional groups modelled in Ecopath were extrapolated to species within the LPI database in order to evaluate the degree to which the indicator captures the complex, ecosystem-wide effects of a policy change [67]. Species were allocated to appropriate functional or taxonomic groups modelled in each ocean system, unless there was no applicable group. There were differences between the level of detail and number of functional groups per model. Some models used functional groups based on taxonomic groups, such as the North Sea model [43], which contains many individual species, sometimes separated by life-history stages, e.g. juvenile and adult Atlantic cod (*Gadus morhua*); other models were more function and habitat focussed, with groups such as benthic coastal invertebrate eaters in the West Florida Shelf model [68]. For example, in the North Sea [43], the humpback whale, *Megaptera novaeangliae*, was allocated to the modelled group “Baleen whale”, and the Atlantic herring, *Clupea harengus*, to the single-species group “Herring”. Where there was more than one model for an ocean system, such as the North Pacific, which contains three models [69], [70], [71], species were allocated to a functional group within the model that best matched the distribution of different populations in the LPI database. Typically only a minority of the groups modelled in Ecopath were represented in the LPI (Table 3).

Thus each model within an ocean system was extrapolated to the level of ocean system by not just allocating species from the model location to a functional group, but allocating most species in the LPI database for the ocean system to a modelled functional group. The LPI uses relative measures of change in abundance of each species as input data; we assumed that changes in modelled biomass as a result of the policy were proportional to changes in abundance. Once all possible species had been allocated a functional group, the projected change in species abundance for each scenario were used to calculate the LPI, a function of the geometric mean of abundance, as described in [16]. Further information on the analyses can be found in [67].

## Supporting Information

Table S1 **Potential source of uncertainty and failure in the indicator-policy cycle, with examples that include retrospective analyses (seeking to understand the previous impacts of policies to inform future action) and prospective analyses (where the impact of alternative actions on both biodiversity and the biodiversity indicators are projected forwards), potential solutions, and those who can address them; numbering relates to points in the cycle shown in** **Figure 1** **.**
(DOCX)

Table S2 **List of all species and key references for estimates of population size and distribution.**
(DOCX)
