# Supplementary material for: Making Robust Policy Decisions Using Global Biodiversity Indicators
Source: PLoS One. 2012 Jul 18;7(7):e41128. doi: 10.1371/journal.pone.0041128 (PMC3399804; doi:10.1371/journal.pone.0041128)
Supplement: Table S1 — Potential source of uncertainty and failure in the indicator-policy cycle, with examples that include retrospective analyses (seeking to understand the previous impacts of policies to inform future action) and prospective analyses (where the impact of alternative actions on both biodiversity and the biodiversity indicators are projected forwards), potential solutions, and those who can address them; numbering relates to points in the cycle shown in Figure 1 . (DOCX) [file pone.0041128.s001.docx]

### Supplementary material

Table S1. Potential source of uncertainty and failure in the indicator-policy cycle, with examples that include retrospective analyses (seeking to understand the previous impacts of policies to inform future action) and prospective analyses (where the impact of alternative actions on both biodiversity and the biodiversity indicators are projected forwards), potential solutions, and those who can address them; numbering relates to points in the cycle shown in Figure 1.

| **Sources of uncertainty and potential failure** | **Examples** | **Solutions** | **Problem solvers** |
| --- | --- | --- | --- |
| 1) Assumptions in the evaluation process | Poor problem definition resulting in unclear goals, badly defined or ambiguous targets | A clear understanding of the problem at hand, clear goals, and a way of translating goals into specific and realistic targets. | Collaboration between policy-makers, stakeholders and scientists |
|  | Uncertainty in models: underestimating or not accounting for key processes (e.g., ecological responses, [1]), the indirect effects of policy (e.g. redistribution of fishing effort [2], or the impacts of other external drivers (e.g. economic forces or climate change [3]) | Strong conceptual frameworks and inter-disciplinary models, with sensitivity analyses to understand the impacts of uncertainties. | Inter-disciplinary scientists in collaboration with subject specialists. |
| 2) Link between evaluation and selection of actions: most appropriate course of action might not be taken because decision-makers have other priorities or the action is unpalatable | Fisheries management: scientific advice on actions such as total allowable catch is often not implemented [4]. | Ensure the set of actions is realistic and can be implemented through stakeholder participation and consultation [5], and improve understanding of the public and decision-makers [6]. | Collaboration between policy-makers, stakeholders and scientists |
| 3) Link between action selection and implementation | Actions may fail to be implemented effectively due to under-resourcing, poor governance or lack of compliance with rules at the local level. e.g. ineffective protected areas [7]. | Improved understanding of human decision making, information on governance and corruption, participatory selection of a set of possible actions to ensure support at implementation [5]. | Social scientists, managers and interdisciplinary scientists |
| 4) Impact of the action differing from the anticipated impact, due to other drivers, indirect effects and externalities | The effects of other drivers obscuring any impact of policy on elephant poaching [3]; poor understanding of the interactions between harvesting and key ecological processes, e.g. the Canadian cod fishery [1]. | Acknowledgement and understanding of types and sources of uncertainty during the evaluation process, leading to better and broader models and improved information | Inter-disciplinary scientists in collaboration with specialists. |
| 5) Link between biodiversity change and indicator change | Poor indicator design renders the indicator a poor proxy for biodiversity [8,9], e.g. protected area coverage provides little indication of the effectiveness of protected areas in conserving biodiversity [10] | More extensive modelling of the behaviour of indicators as the system changes; e.g. extensive testing of indicators in fisheries science [8,11,12,13]; improved suite of indicators to ensure a more structured coverage of aspects of biodiversity change [11]. | Scientists |
|  | Data quality makes trends difficult to detect or interpret due to taxonomic, spatial and temporal gaps and biases [14,15,16,17] | More, and more targeted, data [18], more systematic sampling of taxonomic groups [19], revise indicators to require less data |  |
| 6) Link between indicator change and target assessment: assessing indicators against poorly defined targets, rendering assessment meaningless | Targets are qualitative or ambiguous: e.g. some 2020 CBD target 6; by contrast, Target 11 on protected area coverage is explicit [20]. | Define targets more clearly and in a meaningful way, e.g. SMART targets (specific, measureable, ambitious, realistic and time-bound [21,22]). | Scientists and policy-makers |
|  | Targets are not meaningful biologically (e.g. to avoid tipping points) | Improve understanding of underlying processes [21,22,23,24] | Scientists |
|  | Targets are not compatible, requiring trade-offs between targets. e.g. different groups of species provide different ecosystem services (e.g. climate regulation versus food production versus biodiversity conservation) and may have different management needs [21,25] | Identify potential impacts of alternative policies and achievability of targets through modelling; identify and acknowledge trade-offs, conflicts and synergies between targets and policies [21,26] | Scientists and policy-makers |
| 7) Mismatches in temporal and spatial scales throughout the cycle | Policies decided at a range of scales from global to local; Transboundary effects of national policies on issues such as fisheries management; implementation and data collection occur at the national to local scale. | Identify where spatial mismatches may occur throughout the cycle; Better negotiation and cooperation across borders. | Collaboration between policy-makers, stakeholders and scientists. |
|  | Annual or slower updates of indicators [6] versus decadal changes in major policy such as CBD targets. Ecological processes at a wide range of scales from daily to centuries, including ecological time lags: e.g. extinction debt, time for stock recovery after reduction of fisheries pressure | Explicitly consider processes at different spatial and temporal scales. | Scientists and policy-makers |

### References

1. Petitgas P, Secor DH, McQuinn I, Huse G, Lo N (2010) Stock collapses and their recovery: mechanisms that establish and maintain life-cycle closure in space and time. ICES Journal of Marine Science 67: 1841-1848.

2. Baum JK, Myers RA, Kehler DG, Worm B, Harley SJ, et al. (2003) Collapse and Conservation of Shark Populations in the Northwest Atlantic. Science 299: 389-392.

3. Burn RW (2007) Elephants and Ivory. Significance 4: 118-122.

4. Piet GJ, van Overzee HMJ, Pastoors MA (2010) The necessity for response indicators in fisheries management. ICES Journal of Marine Science 67: 559–566.

5. Levin PS, Fogarty MJ, Murawski SA, Fluharty D (2009) Integrated Ecosystem Assessments: Developing the Scientific Basis for Ecosystem-Based Management of the Ocean. PLoS Biology 7: e1000014.

6. Jones JPG, Collen B, Atkinson G, Baxter P, Bubb P, et al. (2010) The why, what and how of global biodiversity indicators beyond the 2010 Target. Conservation Biology in press.

7. Craigie ID, Baillie JEM, Balmford A, Carbone C, Collen B, et al. (2010) Large mammal population declines in Africa’s protected areas. Biological Conservation 143: 2221-2228.

8. Branch TA, Watson R, Fulton EA, Jennings S, McGilliard CR, et al. (2010) The trophic fingerprint of marine fisheries. Nature 468: 431–435.

9. Grainger A (2008) Difficulties in tracking the long-term global trend in tropical forest area. PNAS 105: 818-823.

10. Chape S, Harrison J, Spalding M, Lysenko I (2005) Measuring the extent and effectiveness of protected areas as an indicator for meeting global biodiversity targets. Philisophical Transactions of the Royal Society B 360: 443-455

11. Fulton EA, Smith ADM, Punt AE (2005) Which ecological indicators can robustly detect effects of fishing? ICES Journal of Marine Science 62: 540-551.

12. Shin Y-J, Shannon LJ, Bundy A, Coll M, Aydin K, et al. (2010) Using indicators for evaluating, comparing, and communicating the ecological status of exploited marine ecosystems. 2. Setting the scene. ICES Journal of Marine Science 67: 692-716.

13. Lindegren M, Mollmann C, Nielsen A, Stenseth NC (2009) Preventing the collapse of the Baltic cod stock through an ecosystem-based management approach. PNAS 106: 14722–14727.

14. Walpole M, Almond REA, Besançon C, Butchart SHM, Campbell-Lendrum D, et al. (2009) Tracking Progress Toward the 2010 Biodiversity Target and Beyond. Science 325: 1503-1504.

15. Collen B, Loh J, Whitmee S, McRae L, Amin R, et al. (2009) Monitoring Change in Vertebrate Abundance: the Living Planet Index. Conservation Biology 23: 317-327.

16. Pereira HM, Cooper HD (2006) Towards the global monitoring of biodiversity change. Trends in Ecology & Evolution 21: 123-129.

17. Magurran AE, Baillie SR, Buckland ST, Dick JM, Elston DA, et al. (2010) Long-term datasets in biodiversity research and monitoring: assessing change in ecological communities through time. Trends in Ecology & Evolution 25: 574-582.

18. Pereira HM, Belnap J, Brummitt N, Collen B, Ding H, et al. (2010) Global biodiversity monitoring. Frontiers in Ecology and the Environment 8: 459–460.

19. Baillie JEM, Collen B, Amin R, Akcakaya HR, Butchart SHM, et al. (2008) Toward monitoring global biodiversity. Conservation Letters 1: 18-26.

20. CoP10 (2010) Conference Of The Parties To The Convention On Biological Diversity: Updating And Revision Of The Strategic Plan For The Post-2010 Period, Nagoya, Japan, 18-29 October. Nagoya, Japan: Convention On Biological Diversity.

21. Perrings C, Naeem S, Ahrestani F, Bunker DE, Burkill P, et al. (2010) Ecosystem Services for 2020. Science 330: 323-324.

22. Mace GM, Cramer W, Diaz S, Faith DP, Larigauderie A, et al. (2010) Biodiversity targets after 2010. Current Opinion in Environmental Sustainability 2: 1–6.

23. Jennings S, Dulvy NK (2005) Reference points and reference directions for size-based indicators of community structure. ICES Journal of Marine Science 62: 397-404.

24. Suding KN, Hobbs RJ (2009) Threshold models in restoration and conservation: a developing framework. Trends in Ecology & Evolution 24: 271-279.

25. Chan KMA, Shaw MR, Cameron DR, Underwood EC, Daily GC (2006) Conservation planning for ecosystem services. PLoS Biology 4: e379.

26. Failing L, Gregory R (2003) Ten common mistakes in designing biodiversity indicators for forest policy. Journal of Environmental Management 68: 121-132.
