# Supplementary material for: Making Robust Policy Decisions Using Global Biodiversity Indicators
Source: PLoS One. 2012 Jul 18;7(7):e41128. doi: 10.1371/journal.pone.0041128 (PMC3399804; doi:10.1371/journal.pone.0041128)
Supplement: Table S2 — List of all species and key references for estimates of population size and distribution. (DOCX) [file pone.0041128.s002.docx]

### Supplementary material

Table S2. List of all species and key references for estimates of population size and distribution

| ***Species name*** | ***Common Name*** | ***Key references*** |
| --- | --- | --- |
| *Acinonyx jubatus* | Cheetah | [1] |
| *Aepyceros melampus* | Impala | [2] |
| *Alcelaphus buselaphus* | Hartebeest | [2] |
| *Antidorcas marsupialis* | Springbok | [2] |
| *Canis simensis* | Ethiopian wolf | [3] |
| *Cephalophus dorsalis* | Bay duiker | [2] |
| *Cephalophus ogilbyi* | Ogilby's duiker | [2] |
| *Cephalophus rufilatus* | Red-flanked duiker | [2] |
| *Cephalophus silvicultor* | Yellow-backed duiker | [2] |
| *Connochaetes gnou* | Black wildebeest | [2] |
| *Connochaetes taurinus* | Common wildebeest | [2] |
| *Damaliscus lunatus* | Tsessebe | [2] |
| *Damaliscus pygargus* | Blesbok/bontebok | [2] |
| *Equus africanus* | African wild ass | [4] |
| *Equus burchellii* | Plains zebra | [4] |
| *Equus grevyi* | Grevy's zebra | [4] |
| *Equus zebra* | Mountain zebra | [4] |
| *Eudorcas rufifrons* | Red-fronted gazelle | [2] |
| *Eudorcas thomsonii* | Thomson's gazelle | [2] |
| *Giraffa camelopardalis* | Giraffe | [2] |
| *Gorilla beringei* | Eastern gorilla | [5] |
| *Hippotragus equinus* | Roan antelope | [2] |
| *Hippotragus niger* | Sable antelope | [2] |
| *Kobus ellipsiprymnus* | Waterbuck | [2] |
| *Kobus kob* | Kob | [2] |
| *Kobus leche* | Southern lechwe | [2] |
| *Kobus vardonii* | Puku | [2] |
| *Litocranius walleri* | Gerenuk | [2] |
| *Lycaon pictus* | African wild dog | [1] |
| *Madoqua guentheri* | Guenther's dik-dik | [2] |
| *Nanger granti* | Grant's gazelle | [2] |
| *Neotragus pygmaeus* | Royal antelope | [2] |
| *Oreotragus oreotragus* | Klipspringer | [2] |
| *Oryx gazelle* | Gemsbok | [2] |
| *Ourebia ourebi* | Oribi | [2] |
| *Pan troglodytes* | Common chimpanzee | [6] |
| *Panthera leo* | Lion | [7] |
| *Pelea capreolus* | Grey rhebok | [2] |
| *Philantomba maxwellii* | Maxwell's duiker | [2] |
| *Raphicerus campestris* | Steenbok | [2] |
| *Raphicerus melanotis* | Cape grysbok | [2] |
| *Redunca arundinum* | Southern reedbuck | [2] |
| *Redunca fulvorufula* | Mountain reedbuck | [2] |
| *Redunca redunca* | Bohor reedbuck | [2] |
| *Sylvicapra grimmia* | Common duiker | [2] |
| *Tragelaphus angasii* | Nyala | [2] |
| *Tragelaphus buxtoni* | Mountain nyala | [2] |
| *Tragelaphus derbianus* | Giant eland | [2] |
| *Tragelaphus eurycerus* | Bongo | [2] |
| *Tragelaphus oryx* | Common eland | [2] |
| *Tragelaphus scriptus* | Bushbuck | [2] |
| *Tragelaphus spekii* | Sitatunga | [2] |
| *Tragelaphus strepsiceros* | Greater kudu | [2] |

### References

1. IUCN/SSC (2007) Regional Conservation Strategy for the Cheetah and African Wild Dog in Eastern Africa. Gland, Switzerland: IUCN Species Survival Commission.

2. East R (1999) African Antelope Database 1998. Gland Switzerland and Cambridge, England: IUCN/SSC Antelope Specialist Group, IUCN (The World Conservation Union).

3. Sillero-Zubiri C, Hoffmann M, Macdonald DW (2004) Canids: Foxes, Wolves, Jackals and Dogs. Status Survey and Conservation Action Plan. Gland, Switzerland and Cambridge, UK: IUCN/SSC Canid Specialist Group.

4. Moehlman PD (2002) Equids: Zebras, Asses, and Horses: Status Survey and Conservation Action Plan. Gland Switzerland and Cambridge, England: IUCN/SCC Equid Specialist Group, IUCN (The World Conservation Union).

5. UNEP World Conservation Monitoring Centre (2003) Report on the status and conservation of the Mountain Gorilla *Gorilla gorilla beringei* Convention on the Conservation of Migratory Species of Wild Animals (CMS), United Nations Environment Programme (UNEP).

6. Kormos R, Boesch C, Bakarr MI, Butynski T, editors (2003) West African Chimpanzees: Status Survey and Conservation Action Plan. Gland, Switzerland: IUCN.

7. Bauer H, Merwe SVD (2004) Inventory of free-ranging lions Panthera leo in Africa. Oryx 38: 26-31.
